# Do Patients with Penetrating Abdominal Stab Wounds Require Laparotomy?

**DOI:** 10.5812/atr.6617

**Published:** 2013-06-01

**Authors:** Behnam Sanei, Mohsen Mahmoudieh, Hamid Talebzadeh, Shahab Shahabi Shahmiri, Zahra Aghaei

**Affiliations:** 1 Department of Surgery, Isfahan University of Medical Sciences, Isfahan, IR Iran

**Keywords:** Anterior Abdominal Stab Wound, Patients, Urgent Laparotomy

## Abstract

**Background:**

The optimal management of hemodynamically stable asymptomatic patients with anterior abdominal stab wounds (AASWs) remains controversial. The goal is to identify and treat injuries in a safe cost-effective manner. Common evaluation strategies are local wound exploration (LWE), diagnostic peritoneal lavage (DPL), serial clinical assessment (SCAs) and computed tomography (CT) imaging. Making a decision about the right time to operate on a patient with a penetrating abdominal stab wound, especially those who have visceral evisceration, is a continuing challenge.

**Objectives:**

Until the year 2010, our strategy was emergency laparotomy in patients with penetrating anterior fascia and those with visceral evisceration. This survey was conducted towards evaluating the results of emergency laparotomy. So, better management can be done in patients with penetrating abdominal stab wounds.

**Patients and Methods:**

This retrospective cross-sectional study was performed on patients with abdominal penetrating trauma who referred to Al- Zahra hospital in Isfahan, Iran from October 2000 to October 2010. It should be noted that patients with abdominal blunt trauma, patients under 14 years old, those with lateral abdomen penetrating trauma and patients who had unstable hemodynamic status were excluded from the study. Medical records of patients were reviewed and demographic and clinical data were collected for all patients including: age, sex, mechanism of trauma and the results of LWE and laparotomy. Data were analyzed with PASW v.20 software. All data were expressed as mean ± SD. The distribution of nominal variables was compared using the Chi-squared test. Also, diagnostic index for LWE were calculated. A two-sided P value less than 0.05 was considered to be statistically significant.

**Results:**

During the 10 year period of the study, 1100 consecutive patients with stab wounds were admitted to Al-Zahra hospital Isfahan, Iran. In total, about 150 cases had penetrating traumas in the anterior abdomen area. Sixty-three (42%) patients were operated immediately due to shock, visceral evisceration or aspiration of blood via a nasogastric tube on admission. Organ injury was seen in 78% of patients with visceral evisceration. Among these 87 cases, 29 patients’ (33.3%) anterior fascia was not penetrated in LWE. So, they were observed for several hours and discharged from the hospital without surgery. While for the remaining 58 patients (66.6%), whose LWE detected penetration of anterior abdominal fascia, laparotomy was performed which showed visceral injuries in 11 (18%) cases.

**Conclusions:**

All in all, 82 percent of laparotomies in patients with penetrated anterior abdominal fascia without visceral evisceration, who had no signs of peritoneal irritation, were negative. So, we recommended further evaluation in these patients. However, visceral evisceration is an indication for exploratory laparotomy, since in our study; the majority of patients had organ damages.

## 1. Background

Nowadays penetrating trauma is increasing because of the growth of violence in our society. There are a large number of patients with penetrating abdominal trauma who have normal vital signs and negative abdominal examination when referred to trauma centers. A great deal of controversy exists between authorities about screening these patients for emergency laparotomy. Although the presence of classic indications such as shock, visceral evisceration and peritoneal irritation suggests laparotomy after penetrating abdominal stab wounds, the trend has moved in the past two decades from mandatory exploration to selective approach (1-5). Mandatory laparotomy for penetrating abdominal stab wounds leads to unnecessary operations in 38–40% of patients, and postoperative morbidity ranges from 3% to 16% (6, 7). Currently there are several possible options for the evaluation of penetrating abdominal trauma in haemodynamically normal trauma patients without signs of peritonitis. Many of these patients will have some superficial tenderness around the wound site, but no signs of peritoneal inflammation. The goal of any algorithm for penetrating abdominal trauma should be to identify injuries requiring surgical repair, and avoid unnecessary laparotomy with its associated morbidity (8-14). The decision as to when to operate on a patient with a penetrating abdominal stab wound is a continuing challenge. Additional diagnostic procedures have been advocated to enhance the sensitivity and specificity of clinical judgment alone in evaluating patients. Several diagnostic methods, including serial physical examination (PE), local wound exploration (LWE), ultrasound (US), computerized tomography (CT), diagnostic peritoneal lavage (DPL), diagnostic laparoscopy (DL), may be used in insignificant injuries on carefully selected patients. LWE is easily and safely performed at the bedside in patients with abdominal stab wounds. The wound is extended under local anesthesia and the track followed through tissue layers (8, 11). If the stab wound tract ends before violation of the abdominal fascia, studies show these patients are safe for discharge. Penetration of the anterior fascia is considered a positive LWE, as penetration of the peritoneum is difficult to identify. A positive LWE leads to either laparotomy or another diagnostic test such as DPL or laparoscopy. When LWE is used alone to determine laparotomy, there will be a high non-therapeutic laparotomy rate. Even if the penetration of peritoneum was used as a cut-off, many of these patients will have no intra-peritoneal injury, or an injury that does not require surgical intervention; most commonly omental laceration, mesenteric laceration or liver tears that have stopped bleeding. On the other hand, exploratory laparotomy for all penetrating abdominal wounds still has a role in resource-limited environments or occasionally in cases of multi-cavitary injuries. For most situations however the non-therapeutic laparotomy rate will be unacceptably high. With the incidence of complications with a negative laparotomy of 12% - 41%, with hospital stays of 4-8 days, it is difficult to support such a strategy where adjunctive methods such as CT and DPL are available and serial physical examination has such a low missed injury rate (15-20). At present, most surgeons believe that the penetration of anterior abdominal fascia in local wound exploration and in the absence of signs of peritoneal irritation, additional diagnostic methods should be carried out. However, in patients with visceral evisceration there is controversy. Although in some trauma centers visceral evisceration is an indication for emergency laparotomy, other centers believe in more diagnostic methods (21-23).

## 2. Objectives

Considering that by the year 2010 in our center, patients with anterior abdominal fascia penetration, and all patients with visceral evisceration are candidates for emergency laparotomy, thus this study has been conducted to evaluate the results of emergency laparotomies to suggest better management of patients with penetrating abdominal stab wounds.

## 3. Patients and Methods

This retrospective cross-sectional study was performed on patients with abdominal penetrating traumas who referred to Al-Zahra hospital in Isfahan, Iran from October 2000 to October 2010. It should be noted that patients with other causes of abdominal trauma, under 14 years old and those with lateral abdomen penetrating trauma were excluded from this study. Indication of urgent laparotomy in our center was patients with visceral evisceration or hemodynamic instability after a penetrating injury to the abdomen, in other patients LWE were first done. If the anterior abdominal fascia was penetrated urgent laparotomy without any further diagnostic modalities was done. Medical records of patients were reviewed. Demographic and clinical data were collected for all patients including: age, sex, mechanism of trauma and the results of LWE and laparotomy. As an initial approach, the standard resuscitative protocol, approved by the advanced trauma life support guidelines, was used for patient care. Preoperative antibiotics such as cefozoline sodium (20 mg/kg) were administered intravenously, and all patients received tetanus toxoid. Nasogastric tube and Foley catheter were inserted in selected patients. Wound exploration was performed to wounds inferior to the costal margins and between the mid-axillary lines. Patients with stab wounds in other locations such as the back were evaluated separately. Following skin preparation with an antiseptic solution, a local anesthetic agent was applied to the wound. A retractor was inserted through the entry of the wound and peritoneal or posterior fascia penetrations were investigated (8). In our study if anterior fascia was penetrated without any further diagnostic methods, urgent laparotomy was done. Data were analyzed using PASW (Predictive Analysis Software ) v.18 software. All data were expressed as mean ± SD. The distribution of nominal variables was compared using the Chi-squared test. Also, diagnostic index for LWE were calculated. A two-sided P < 0.05 was considered to be statistically significant.

## 4. Results

**Figure 1.**
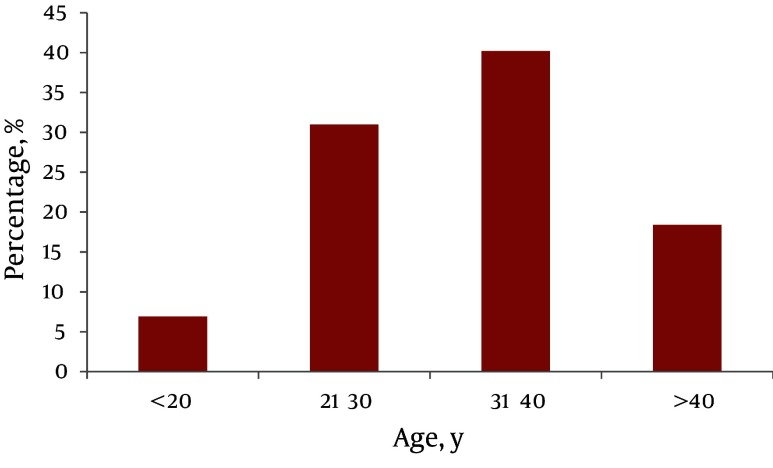
Age Distribution of Patients with Anterior Abdominal Stab Wounds

During the 10 year period of study, 1100 consecutive patients with stab wounds were admitted to Al-Zahra hospital. In total, about 150 cases had penetrating traumas to the anterior abdomen area (Figure 1). Sixty-three (42%) patients who were operated immediately due to shock, irradiation of organs, or aspiration of blood via the nasogastric tube on admission were excluded from the trial. Of these patients, nineteen (17%) cases for unstable hemodynamic, two (3%) cases for aspiration of blood via the nasogastric tube were operated immediately. In 29 cases (33.3%), the results of LWE and laparotomy similarly showed organ damage. However, in 37 cases the results of LWE and laparotomy were not the same (P < 0.001). A sensitivity of 100%, specificity of 38.18%, positive predictive value (PPV) of 18.96%, negative predictive value (NPV) of 100% and positive likelihood ratio (PLR) of 1.61, were found in diagnosing visceral injury for LWE (Table 1). Of the remaining, Forty-two (80%) patients with penetration of peritoneum, 30 patients had eviscerated omentum and 12 had eviscerated small bowel where laparotomy showed visceral injury in 33 (78%) cases. For the remaining 87 (58%) patients with stab wounds, penetrating of the peritoneum LWE was done. Among these 87 cases, in 29 patients (33.3%) anterior fascia was not penetrated in LWE, so they were observed for several hours and discharged from the hospital without surgery, while for the remaining 58 patients (66.6%), for whom LWE found penetration of anterior abdominal fascia, laparotomy was performed. Only in 11 (18%) cases visceral injuries has been reported.

**Table 1.** The Results of Local Wound Exploration and Laparatomy in Diagnosis of Visceral Injury in Patients with Stab Wounds

LWE ^a^	OR ^b^ Positive, No.	OR ^b^ , Negative, No.	OR ^b^ , Total, No.
**Positive**	11	47	58
**Negative **	0	29	29
**Total**	11	76	87

^a^Abbreviation: LWE, local wound exploration; ^b^ OR, laparatomy

## 5. Discussion

Which diagnostic tree a hospital chooses for the evaluation of penetrating injuries will be dependent on numerous factors, including trauma patient load, surgical team availability and coverage, the availability of multidetector CT scanners and trauma radiologists, and access to the operating room and critical care beds. Many different systems are used around the world. The following recommendations are in order of preference and are by no means the only possibilities. Each choice is associated with serial physical examination, multidetector CT, local wound exploration, diagnostic peritoneal lavage or laparoscopy. Although the rate of nontherapeutic laparotomies after penetrating wounds to the abdomen should be minimized, this should never be at the expense of a delay in the diagnosis and treatment of the injury. With this in mind, a routine laparotomy is not indicated in hemodynamically stable patients with abdominal stab wounds without signs of peritonitis or diffuse abdominal tenderness. The vast majority of patients with penetrating abdominal trauma managed nonoperatively may be discharged after twenty-four hours of observation in the presence of a reliable abdominal examination and minimal to no abdominal tenderness. LWE has been used in a number of series to rule out penetration of the anterior fascia; if a patient has no penetration of the anterior fascia, the patient may be safely discharged from the emergency department. Most authors, however, have investigated only the anterior fascia when performing LWE (8). Mandatory laparotomy after anterior fascial penetration has been reported as negative in almost 50%, so further diagnostic testing to rule out intraperitoneal injuries requiring operation is a better option than mandatory laparotomy. Clarke et al. (2) evaluated 340 patients with a penetrating anterior abdominal stab wound who were managed over a two-year period. A total of 192 (56%) of these patients were subjected to mandatory laparotomy. Of these mandatory laparotomies, 98% were positive. Thirty (20%) out of the remaining 148 (44%) observed patients underwent surgery subsequently. A total of 13 patients were only taken to surgery after 12 hours of observation. They concluded that clinical assessment accurately predicts the need for mandatory laparotomy following a stab wound to the torso (2). In another study by Navasaria and colleague, they assessed 186 patients with abdominal stabs where seventy-four patients (39.8%) underwent emergency laparotomy. There were 5 negative laparotomies (6.8%). The remaining 112 patients (60.2%) were assigned for abdominal observation. One hundred (89.3%) of these patients were successfully managed non-operatively. The remaining 12 patients underwent delayed laparotomy, which was negative in two cases (16.7%). Non-operative management was successful in 53.8% of patients overall. The overall sensitivity and specificity of serial abdominalexamination was 87.3% and 93.5%, respectively. They concluded that serial physical examination alone for asymptomatic or mildly symptomatic patients with abdominal stab wounds enables a significant reduction in unnecessary laparotomies (5). We found that the rate of visceral injuries was just 18 percent in the patients with penetrating anterior fascia, although there was no evidence of visceral evisceration. Similarly, results of other studies showed that it is necessary to do more diagnostic modalities in patients with penetrated anterior abdominal fascia in local wound exploration, despite the absence of signs of peritoneal irritation and visceral evisceration (1, 2, 4, 5, 7, 12, 14, 18). The diagnostic values observed for LWE in our study were in agreement with other researches. There is a great deal of controversy surrounding authorities over emergency laparotomy in patients with visceral evisceration. Some investigators suggest selective observation in these cases (8, 24, 25). However, many reports have shown that visceral evisceration is an indication for emergency laparotomy because the rate of organ injuries is about 70 - 80 percent (1, 3, 15, 18-21, 26, 27). Results of our study showed that forty-two patients had penetration of peritoneum; 30 (71%) patients had eviscerated omentum, and 12 (29%) had eviscerated small bowel. Laparotomy showed visceral injury in 33 (78%) cases, thus emergency intervention seems prudent. In summary, in patients with penetrated anterior abdominal fascia without visceral evisceration and in the absence of signs of peritoneal irritation, rate of negative laparatomy was 82 percent. We recommend the identification of criteria for indication of urgent laparatomy, and performance of laparatomy only in the selected cases. However, visceral evisceration is an indication for exploratory laparotomy, since majority of patients had organ damages in our study.

## References

[1] Butt MU, Zacharias N, Velmahos GC (2009). Penetrating abdominal injuries: management controversies.. Scand J Trauma Resusc Emerg Med..

[2] Clarke DL, Allorto NL, Thomson SR (2010). An audit of failed non-operative management of abdominal stab wounds.. Injury..

[3] da Silva M, Navsaria PH, Edu S, Nicol AJ (2009). Evisceration following abdominal stab wounds: analysis of 66 cases.. World J Surg..

[4] Nair MS, Uzzaman MM, Al-Zuhir N, Jadeja A, Navaratnam R (2011). Changing trends in the pattern and outcome of stab injuries at a North London hospital.. J Emerg Trauma Shock..

[5] Navsaria PH, Berli JU, Edu S, Nicol AJ (2007). Non-operative management of abdominal stab wounds--an analysis of 186 patients.. S Afr J Surg..

[6] Demetriades D, Hadjizacharia P, Constantinou C, Brown C, Inaba K, Rhee P (2006). Selective nonoperative management of penetrating abdominal solid organ injuries.. Ann Surg..

[7] Inaba K, Demetriades D (2007). The Nonoperative Management of Penetrating Abdominal Trauma.. Adv Surg..

[8] Arikan S, Kocakusak A, Yucel AF, Adas G (2005). A prospective comparison of the selective observation and routine exploration methods for penetrating abdominal stab wounds with organ or omentum evisceration.. J Trauma..

[9] Beekley AC, Blackbourne LH, Sebesta JA, McMullin N, Mullenix PS, Holcomb JB (2008). Selective nonoperative management of penetrating torso injury from combat fragmentation wounds.. J Trauma..

[10] Chiu WC, Shanmuganathan K, Mirvis SE, Scalea TM (2001). Determining the need for laparotomy in penetrating torso trauma: a prospective study using triple-contrast enhanced abdominopelvic computed tomography.. J Trauma..

[11] Morrison JJ, Dickson EJ, Jansen JO, Midwinter MJ (2012). Utility of admission physiology in the surgical triage of isolated ballistic battlefield torso trauma.. J Emerg Trauma Shock..

[12] Pryor JP, Reilly PM, Dabrowski GP, Grossman MD, Schwab CW (2004). Nonoperative management of abdominal gunshot wounds.. Ann Emerg Med..

[13] Shan CX, Ni C, Qiu M, Jiang DZ (2012). Is laparoscopy equal to laparotomy in detecting and treating small bowel injuries in a porcine model?. World J Gastroenterol..

[14] Wiewiora M, Sosada K, Piecuch J, Zurawinski W (2011). The role of laparoscopy in abdominal trauma - review of the literature.. Wideochir Inne Tech Malo Inwazyjne..

[15] Bautz PC (2007). Management of stab wounds in South Africa.. ANZ J Surg..

[16] Haan J, Kole K, Brunetti A, Kramer M, Scalea TM (2003). Nontherapeutic laparotomies revisited.. Am Surg..

[17] Leppaniemi A, Haapiainen R (2003). Diagnostic laparoscopy in abdominal stab wounds: a prospective, randomized study.. J Trauma..

[18] Sugrue M, Balogh Z, Lynch J, Bardsley J, Sisson G, Weigelt J (2007). Guidelines for the management of haemodynamically stable patients with stab wounds to the anterior abdomen.. ANZ J Surg..

[19] Van Brussel M, Van Hee R (2001). Abdominal stab wounds: a five-year patient review.. Eur J Emerg Med..

[20] van Haarst EP, van Bezooijen BP, Coene PP, Luitse JS (1999). The efficacy of serial physical examination in penetrating abdominal trauma.. Injury..

[21] Practice management guidelines for nonoperative management of penetrating abdominal trauma.. http://www.east.org.

[22] Soto JA, Morales C, Munera F, Sanabria A, Guevara JM, Suarez T (2001). Penetrating stab wounds to the abdomen: use of serial US and contrast-enhanced CT in stable patients.. Radiology..

[23] Stockinger ZT, McSwain NE, Jr (2004). Prehospital endotracheal intubation for trauma does not improve survival over bag-valve-mask ventilation.. J Trauma..

[24] Huizinga WK, Baker LW, Mtshali ZW (1987). Selective management of abdominal and thoracic stab wounds with established peritoneal penetration: the eviscerated omentum.. Am J Surg..

[25] McFarlane ME (1996). Non-operative management of stab wounds to the abdomen with omental evisceration.. J R Coll Surg Edinb..

[26] Burnweit CA, Thal ER (1986). Significance of omental evisceration in abdominal stab wounds.. Am J Surg..

[27] Nagy K, Roberts R, Joseph K, An G, Barrett J (1999). Evisceration after abdominal stab wounds: is laparotomy required?. J Trauma..

